# Society Meetings

**Published:** 1908-10

**Authors:** 


### SOCIETY MEETINGS.

The Medical Association of Central New York will hold its forty-first annual meeting at Buffalo, Tuesday October 20, 1908, in the auditorium of the Historical Society's building, Delaware park. The morning session will open at ten o'clock, and the afternoon session at two o'clock. A program has been proposed by the officers and the following papers will be presented:

President's Address, Carlton C. Frederick, Buffalo; Diet in Typhoid Fever, I. H. Levy, Syracuse; The Surgical Idea in Obstetrics, Wm. Mortimer Brown, Rochester; Five Cases of Spinal Cord Tumor with Operations, Wm. C. Krauss, Buffalo; A Surgical Evening in Italy, Lewis A. Rose, Rochester; County Sanitary Associations and their Uses, O. J. Hallenbeck, Canandaigua; Senility—Its Symptoms, Approach and Treatment, J. Moyer Hagey, Mt. Morris; Guesswork in the Practice of Medicine, B. C. Loveland, Syracuse; Some Points in Abdominal Diagnosis, W. W. Skinner, Geneva; Further Research Regarding the Phosphatic Index or the Pulse of the Nervous System, J. Henry Dowd, Buffalo; Renal Decapsulation for Eclampsia, Wm. B. Jones, Rochester; Endothelioma of Bone Following Injury, G. W. Cottis, Batavia; Complications and Sequelæ, I. M. Slingerland, Fayetteville; Blastomycosis in New York State, Henry Clay Baum, Syracuse; A Case of Dermatitis Exfolativa, Wm. A. Howe, Phelps; Chronic Cholocystitis with its Complications and Sequelæ, H. M. Spofford, Batavia; The Effect of High Frequency Currents upon Blood-pressure; Demonstration of D. Arsonval Cage, W. L. Conklin, Dansville; Fracture and Dislocation of Shoulder with History and Specimens of Case, D. M. Totman, Syracuse; The Prognosis in Achylea Gastrica, Allen A. Jones, Buffalo; A Few Clinical Reports, Henry L. Elsner, Syracuse; A New Method of Treatment of Chancroid by Means of Hot Air, E. Wood Ruggles, Rochester; Treatment of Eclampsia, W. L. Wallace, Syracuse; The Etiology of Chronic Deafness, W. Scott Renner, Buffalo.

The officers of the association are: president, C. C. Frederick, Buffalo; first vice-president, M. P. Conway, Auburn; second vice-president, A. A. Young, Newark; secretary, C. A. Greenleaf, Rochester; treasurer, Wm. M. Brown, Rochester. The local committee on arrangements are, William C. Krauss, chairman; A. A. Hubbell, L. Howe, C. G. Stockton and F. S. Crego.

The association covers all the western and central New York counties and has achieved a reputation for scientific work as evidenced by its annual volume of transactions. Physicians in general are cordially invited to attend the meetings.

---

The American Dermatological Association held its regular annual meeting at Annapolis and Baltimore, Md., September 24, 25 and 26, 1908, when the following officers were elected for the ensuing year: president, William Thomas Gilcrist, of Baltimore; vice-president, William Allen Pusey, of Chicago; secretary and treasurer, Grover William Wende, of Buffalo. The next meeting of the association is to be held at Philadelphia, in May or June, 1909.

---

The Association of Erie Railway Surgeons will hold its seventeenth annual meeting at the Hotel Statler, Buffalo, October 7 and 8, 1908. Several prominent members are listed for papers and a banquet will be held at The Statler on the evening of the second day. Dr. W. H. Mansperger of Buffalo has arranged clinics at the German Deaconess's Hospital on October 8th and there will be a special trolley trip to Niagara Falls and around the Gorge route, as well as an automobile ride about Buffalo.

---

The American Association of Obstetricians held its twenty-first annual meeting at Baltimore, September 22, 23, and 24, 1908, during which the following named officers were elected to serve until their successors are chosen: president, William Henry Humiston; vice-presidents, James E. Sadlier, Poughkeepsie, and John D. S. Davis, Birmingham; secretary, William Warren Potter, Buffalo (re-elected); treasurer, X. O. Werder, Pittsburg (re-elected); executive councillors, E. Gustav Zinke, Cincinnati, and Herman E. Hayd, Buffalo; also executive councillor, to fill vacancy occasioned by the election of William H. Humiston to the presidency, William A. B. Sellman, Baltimore. Next place of meeting, Fort Wayne, Ind., the 3d Tuesday in September (21), 1909.

---

THE third annual meeting of the eighth district branch of the Medical Society of the State of New York, was held at Batavia. September 22 and 23, 1908. E. E. Snow of Batavia presided. Papers were read by Drs. DeLancey Rochester and H. C. Rooth, T. H. McKee, C. A. Wall, N. G. Russell, James A. Gardner, James E. King, William C. Krauss, James W. Putnam and H. R. Hopkins of Buffalo.

The following officers were elected: president, E. E. Snow of Batavia; vice-presidents, Edward Munson of Medina and T. H. McKee of Buffalo; secretary, Lee M. Francis of Buffalo, and treasurer, C. A. Wall of Buffalo.

